# Effectiveness of Xiakucao Oral Liquid combined with methimazole on early thyroid function recovery and treatment compliance in Graves’ disease: a real-world retrospective cohort study

**DOI:** 10.3389/fendo.2025.1560262

**Published:** 2025-06-06

**Authors:** Dong Lin, Lina Zhang, Lu Zhang, Jialu Zhu, Houcheng Li, Bichai Yuan

**Affiliations:** ^1^ Department of Neurology, Jieyang People’s Hospital, Jieyang, China; ^2^ The Second Medical Department, Shanghai Haitian Pharmaceutical Technology Development Co., Ltd., Shanghai, China; ^3^ School of Public Health, Fudan University, Shanghai, China; ^4^ Department of Endocrinology, Jieyang People’s Hospital, Jieyang, China

**Keywords:** Graves’ disease, Xiakucao Oral Liquid, methimazole, sensitive thyroid-stimulating hormone, real-world

## Abstract

**Aim:**

To evaluate the effectiveness and treatment compliance of Xiakucao Oral Liquid (XKC) combined with methimazole (MMI) on the early recovery of thyroid function in Graves’ disease (GD) patients.

**Methods:**

In this retrospective cohort study, GD patients receiving initial treatment at Jieyang People’s Hospital from January 2019 to December 2023 were included and divided into XKC+MMI group and MMI group based on the treatment regimen. The association between XKC+MMI and early thyroid function recovery was analyzed using multiple linear regression in the subgroup of patients with post-treatment data available at 3 months.

**Results:**

A total of 441 patients were enrolled, among whom 113 (25.6%) patients received XKC+MMI. The proportion of patients receiving XKC+MMI was significantly higher with an initial MMI dose ≥20 mg/day than <20 mg/day (37.1% *vs*. 15.2%, P=0.006). In XKC+MMI group, 15 (13.3%) patients underwent continuous treatment with XKC for three months. Subgroup analysis (n=121) showed the increase of sensitive thyroid-stimulating hormone (sTSH) in XKC+MMI group was higher than that in MMI group (4.86 ± 11.7 *vs* 1.15 ± 5.16). Multivariate linear regression analysis indicated that compared with MMI alone, XKC+MMI was independently associated with the increase of sTSH (β=3.346, 95%CI: 0.353-6.339, P=0.031), and the continuous 3-month treatment of XKC+MMI was also independently associated with the increase of sTSH at 3 months (β=4.062, 95%CI: 0.516-7.608, P=0.027).

**Conclusions:**

XKC combination MMI treatment might promote early recovery of thyroid function. However, adherence to and persistence with XKC treatment still need to be improved.

## 1 Introduction

Graves’ disease (GD) is the most common type of hyperthyroidism, accounting for about 80% of hyperthyroidism (1). GD is an autoimmune disease characterized by immune system dysregulation that causes the produce of thyroid-stimulating hormone (TSH) receptor antibodies (TRAb). These antibodies activate the TSH receptor to promote the synthesis and excessive secretion of thyroid hormones, resulting in hyperthyroidism and harm to multiple organs and systems of the body (2). The clinical manifestations include diffuse goiter, Graves ophthalmopathy, cutaneous mucous lesions and hyperthyroidism acromegaly. The incidence of GD is approximately 2.0-3.0% in China and around 0.5-2.0% in Western countries (3).

GD has been demonstrated to be associated with a significantly increased risk of cardiovascular events and all-cause mortality (4–7). Notably, the mortality rate and the risk of acute cardiovascular events peak in the first 3 months after diagnosis of hyperthyroidism (8). Large-sample real-world studies have demonstrated that the longer the duration of hyperthyroidism in GD patients, especially the longer the duration of low TSH level, the higher the risk of developing dementia (9), cardiovascular disease (10) and death (6, 8, 11). Moreover, sustained elevation of TRAb levels not only increase the risk of Graves’ ophthalmopathy (12, 13) but also lead to adverse pregnancy outcomes and neonatal thyroid dysfunction (14, 15). Therefore, effective treatment regimens for GD patients, especially those that facilitated an early recovery of TSH levels, are of great significance for improving patient prognosis.

Methimazole (MMI), recognized as the preferred drug for antithyroid drug (ATD) therapy, has been found to normalize the thyroid function in only 37% of GD patients with a dosage of 10 mg/day of MMI for one month (16). Studies have shown that the MMI cannot normalize the thyroid function in some GD patients, especially in terms of early recovery to normal levels (17, 18). Furthermore, 12–18 months of ATD treatment only results in the remission of hyperthyroidism in 30-50% of patients (19–21). Study also indicated that only 46.51% of GD patients can return to normal TSH levels after 9 months of ATD treatment (22). Therefore, more treatment options are needed to help GD patients recover thyroid function to normal levels more quickly.

Many studies have demonstrated the benefits of Chinese patent medicine combined with MMI in the treatment of GD, including shortening treatment course, reducing thyroid volume, alleviating symptoms and lessening adverse reactions (23–28). Xiakucao Oral Liquid (XKC), manufactured by Guiyang Xintian Pharmaceutical Co., LTD (National Drug Approval No. Z19990052), is used for the treatment of headaches, dizziness, goiters, lymphadenitis, thyroid nodules, breast abscesses accompanied by pain, enlarged thyroid gland, and breast hyperplasia in clinical practice. Clinical trials have demonstrated that the combination of XKC with MMI (XKC+MMI) in the treatment of GD patients can better relieve symptoms, reduce goiter, improve thyroid hormone levels and decrease adverse reactions compared to MMI monotherapy (23, 24, 29, 30). In addition, this combination therapy has been shown to significantly reduce the time required to improve thyroid function (24). However, no studies have investigated the association between XKC+MMI and the early recovery of thyroid function in GD patients in clinical practice. Moreover, there is also a lack of research on the real-world clinical application of XKC.

This retrospective study analyzed data from real clinical practice, aimed to explore the treatment compliance of XKC and the association between XKC+MMI and the early recovery of thyroid function in GD patients.

## 2 Methods

### 2.1 Study design and participants

This retrospective cohort study was conducted in the Department of Endocrinology of Jieyang People’s Hospital. Data of GD patient who visited the hospital from January 2019 to December 2023 were collected from the electronic medical record system. The inclusion criteria were: 1) aged 18 to 75 years, 2) clinically diagnosed with GD, 3) receiving the initial treatment in Department of Endocrinology, 4) treated with MMI or XKC+MMI. The diagnosis of GD was confirmed based on the presence of both radiographic evidence and clinical diagnosis (31). The exclusion criteria were: 1) without baseline data such as age, sex, and thyroid function indicators, 2) without laboratory test records except for the baseline. This study was approved by the Clinical Research Ethics Committee of Jieyang People’s Hospital (Ethical approval number: 2024006), and informed consent was waived by the committee because of the retrospective nature of the study. This study was registered in the International Traditional Medicine Clinical Trials Registration, registration number: ITMCTR2024000684.

### 2.2 Group and treatment

Participants were grouped into XKC+MMI group and MMI group according to treatment regimen.

Participants in the MMI group received monotherapy with MMI. In clinical practice, the dosage range of MMI is 5–40 mg per day. The dosage is determined on an individual basis according to the severity of the patient’s disease and thyroid function indicators.

Participants in the XKC + MMI group were treated with the MMI regimen plus XKC. XKC is a herbal preparation made from the Prunella vulgaris. Each 10 ml of XKC is equivalent to 8 grams of raw Prunella vulgaris. The use of XKC was determined through shared decision-making between physicians and patients. XKC was administered at a fixed dose of 10 mL twice daily (bid) for all adult patients in the XKC+MMI group. Treatment adherence was monitored through a combination of pharmacy dispensing records.

### 2.3 Data collection and definition

Data was obtained from the electronic medical record system. Data collected for analysis included demographics (age and sex), sensitivity thyroid-stimulating hormone (sTSH, normal range: 0.27-4.2 μIU/mL), free triiodothyronine (FT3, normal range: 3.1-6.8 pmol/L), free thyroxine (FT4, normal range: 12.3-20.2 pmol/L), TRAb (normal range: 0-1.75 IU/L), MMI doses and the duration of XKC treatment.

The outcomes were 3 months changes of sTSH and TRAb post-treatment. The 3 months thyroid function was analyzed using the laboratory test results between 2.5 and 3.5 months after the baseline. The treatment duration of XKC refers to the total time a patient receives XKC treatment, extracted from prescription information, and may involve either continuous or intermittent treatment.

Participants with thyroid function laboratory tests at the third month were included in the subgroup to analyze the association between the treatment regimen and outcomes.

### 2.4 Statistical analysis

SPSS 24.0 (IBM, Armonk, NY, USA) and R (version 4.4.2) software (https://www.r-project.org/) were used for statistical analysis. The Kolmogorov-Smirnov (K-S) test was used to assess whether continuous variables conform to a normal distribution. Continuous variables with a normal distribution were presented as means ± standard deviations. Continuous variables that do not conform to a normal distribution were presented as median (minimum, maximum). Comparisons between groups of continuous variables conforming to a normal distribution were assessed with Student’s t-test. The Mann-Whitney U test was used to compare continuous variables that did not conform to normal distribution between groups.

In subgroup, variable with missing data were FT3 and TRAb. Missing data were handled using multiple imputation by chained equations (MICE package) in R. This approach accounts for the uncertainty of missing values by generating multiple plausible imputed datasets, which were subsequently pooled for analysis to reduce bias compared to single imputation or complete-case analysis. The imputation model included all variables used in the final analysis (e.g., age, sex, baseline sTSH and FT4 levels, treatment group). Continuous variables were imputed using predictive mean matching, while binary variables were imputed based on the range of continuous variables. A sensitivity analysis was conducted in subgroup by repeating the primary analysis under the missing-at-random assumption and comparing results with those from complete-case analysis. The consistency between imputed and complete-case estimates supported the robustness of our findings.

Two multiple linear regression models were used to adjust for potential confounding factors and assess the independent effect of XKC and XKC duration. Interaction terms were further performed to evaluate whether the effect of treatment varied across subgroups defined by MMI dose. All independent variables were entered into the multivariate model using the enter method. P<0.05 was statistically significant.

A *post hoc* power analysis was conducted to estimate the statistical power of detecting the observed between-group difference in TRAb reduction (MMI group: -3.94 ± 5.97, MMI+XKC group: -1.06 ± 6.00) under the current sample size. Using a two-sided independent t-test with a significance level (α) of 0.05 and the actual sample sizes of 32 participants in the XKC+MMI group and 89 in the MMI group, the achieved power was calculated as 63.2%. This indicates that the study had not sufficient power (<80%) to detect a clinically meaningful difference in TRAb levels.

## 3 Results

### 3.1 Baseline characteristics

**Figure 1:**
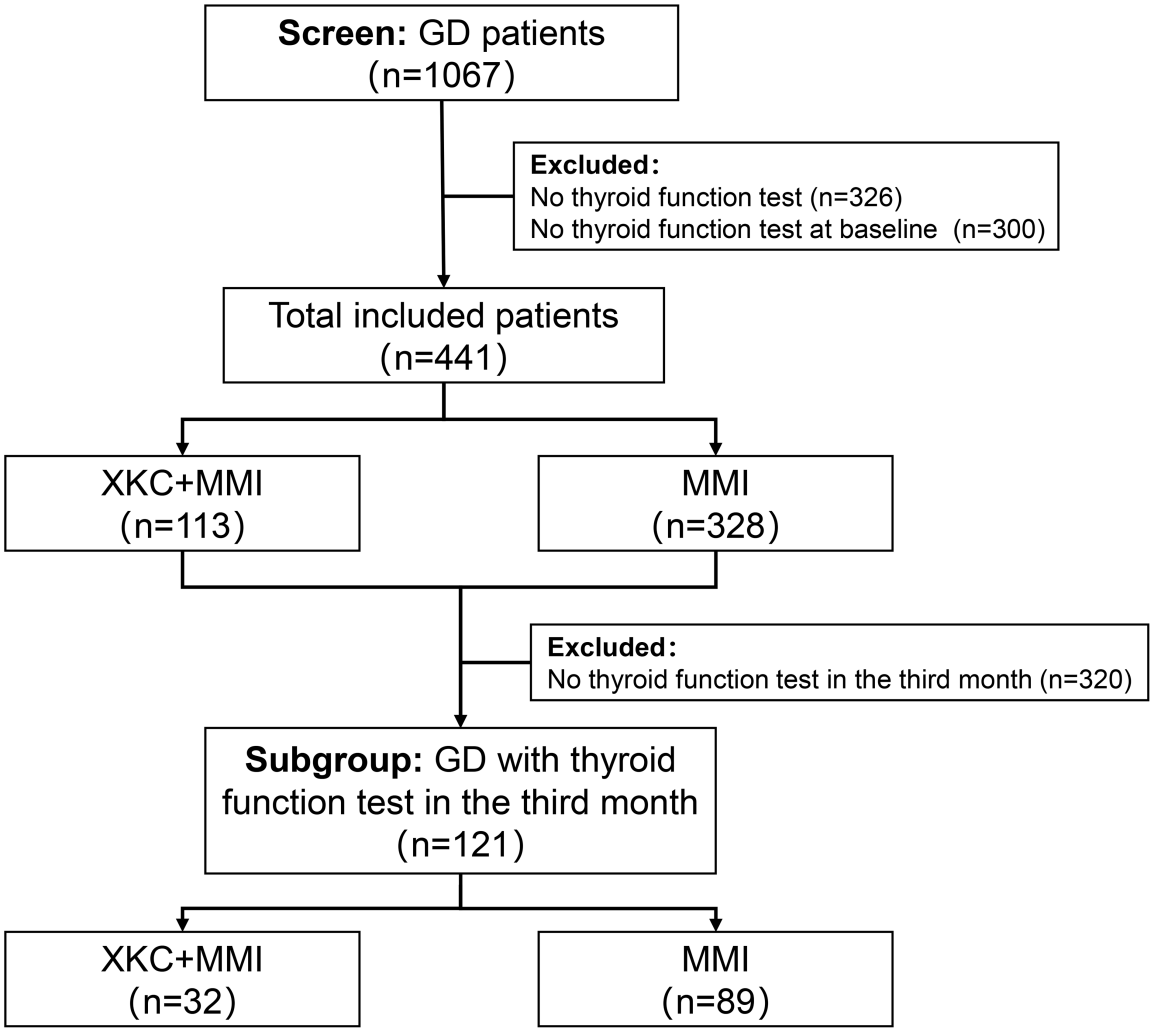
The flow chart of the study.

**Figure 2:**
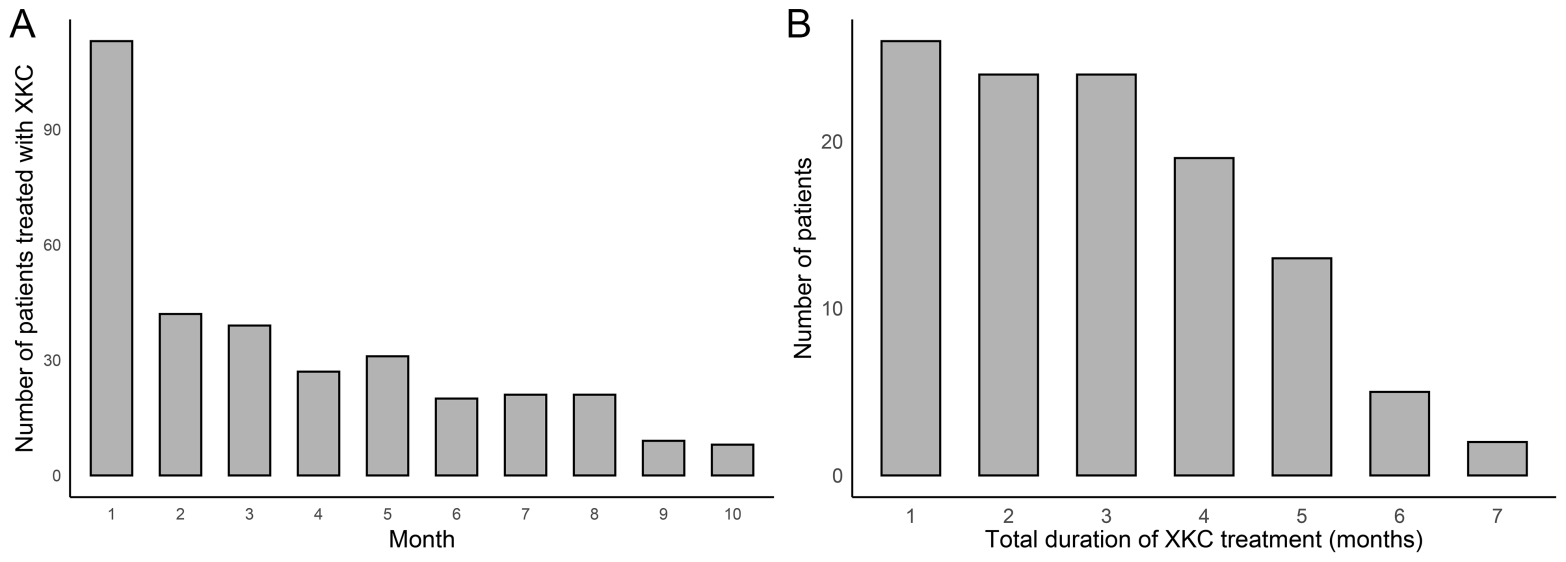
The clinical application of XKC (n=441). **(A)** Number of patients treated with XKC per month after baseline. **(B)** Cumulative duration of XKC treatment.

After screening, a total of 1067 GD patients visited outpatient clinics or were hospitalized, among whom 264 (24.7%) received treatment with XKC. Excluding patients with missing thyroid function data and those who were not first-time visitors to the hospital, a total of 441 patients were enrolled in the study, including 334 (75.7%) females and 107 (24.3%) males. **Figure 1** shows the flow chart of the study. A total of 113 (25.6%) patients received treatment with the combination of XKC+MMI, and 328 (74.4%) received MMI. The percentage of patients with suppressed sTSH levels below the normal range was higher in patients treated with XKC+MMI than that of patients treated with MMI alone (81.4% *vs* 69.5%, P=0.01) (**Table 1**). At baseline, a total of 113 patients received XKC treatment. One month later, the number decreased to 42, and the number of patients receiving XKC decreased month by month (**Figure 2A**). There were 26, 24 and 24 patients who received XKC treatment for a cumulative duration (discontinuous or continuous) of 1 month, 2 months and 3 months respectively. Only 5 patients received XKC treatment for up to 6 months. (**Figure 2B**). In the XKC+MMI group, only 15 (15/113, 13.3%) patients continuously received XKC for 3 months.

**Table 1:** Baseline characteristics of GD patients (n=441).

Variables	XKC+MMI (n=113)	MMI (n=328)	P
Male, n (%)	28 (24.8)	79 (24.1)	0.996
Age, year, mean±SD	38.18±12.07	40.08±13.36	0.258
FT3, pmol/L			
N (N missing)	109 (4)	320 (8)	
mean±SD	13.63±12.21	11.76±10.51	0.155
Median (Q1, Q3)	8.03 (5.26, 17.64)	7.14 (4.90, 13.39)	
FT3, n (%)			0.528
Normal range	44 (38.9)	142 (43.3)	
Below normal	1 (0.01)	5 (0.02)	
Above normal	64 (56.6)	173 (52.7)	
Missing	4 (0.4)	8 (2.4)	
FT4, pmol/L			
N (N missing)	113 (0)	328 (0)	
mean±SD	35.39±29.08	31.94±26.23	0.544
Median (Q1, Q3)	20.51 (14.57, 45.37)	21.03 (14.72, 38.87)	
FT4, n (%)			0.715
Normal range	42 (37.2)	117 (35.7)	
Below normal	12 (10.6)	43 (13.1)	
Above normal	59 (52.2)	168 (51.2)	
sTSH, uIU/L			
N (N missing)	113 (0)	328 (0)	
mean±SD	0.61±2.46	1.22±4.92	**0.021**
Median (Q1, Q3)	0.005 (0.005, 0.027)	0.005 (0.005, 0.829)	
sTSH, n (%)			**0.010**
Normal range	19 (16.8)	77 (23.5)	
Below normal	92 (81.4)	228 (69.5)	
Above normal	2 (1.8)	23 (7.0)	
TRAb, IU/L			
N (N missing)	104 (9)	300 (28)	
mean±SD	15.40±12.03	12.57±11.03	**0.037**
Median (Q1, Q3)	11.22 (5.21, 24.81)	8.27 (4.15, 18.29)	
TRAb, n (%)			0.326
Normal range	4 (3.5)	23 (7.0)	
Above normal	100 (88.5)	277 (84.5)	
Missing	9 (8.0)	28 (8.5)	
Initial dose of MMI, mg/d, n (%)			**<0.001**
<20	53 (46.9)	217 (66.2)	
≥20	60 (53.1)	111 (33.8)	
Duration of XKC administration, month, n (%)			
1-2	50 (44.2)	–	
3	24 (21.2)	–	
4	19 (16.8)	–	
≥5	20 (17.7)	–	

XKC, Xiakucao Oral Liquid; MMI, Methimazole; sTSH, sensitive thyroid-stimulating hormone; TRAb, thyroid-stimulating hormone receptor antibody; FT3, free triiodothyronine; FT4, free thyroxine.

Bold values indicate P<0.05.

### 3.2 The subgroup with thyroid function tested three months after medication

**Figure 3:**
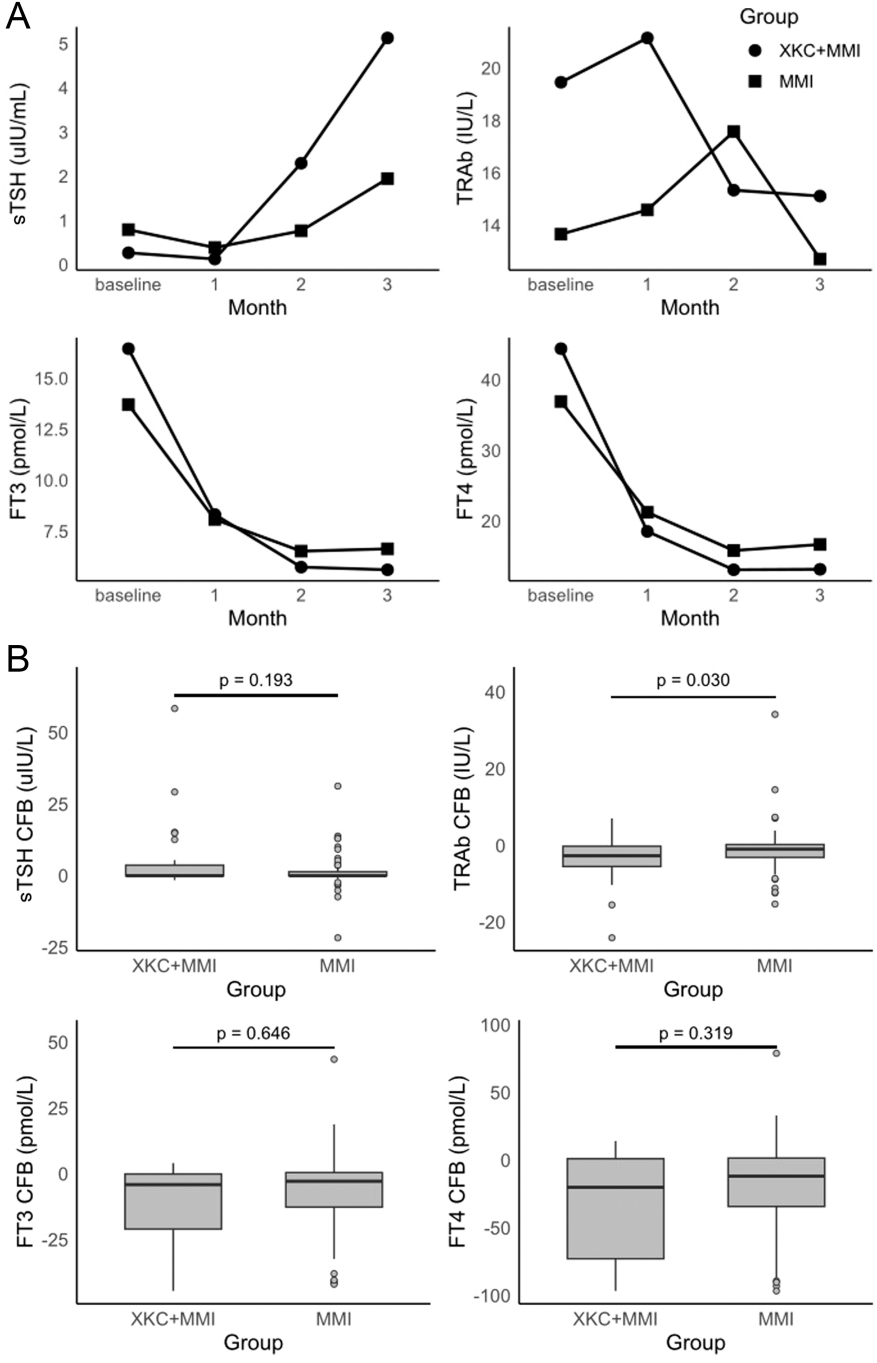
**(A)** time-specific absolute values of thyroid hormone, and **(B)** changes of thyroid hormones at 3 months from baseline in subgroups (not adjusted analysis) (n=121, XKC+MMI: 32, MMI:89).

A total of 121 patients were in the subgroup, including 32 patients (32/121, 26.4%) in the XKC+MMI group and 89 patients (89/121, 73.6%) in the MMI group (**Table 2**). The percentage of patients receiving XKC+MMI was significantly higher in the subgroup with an initial dosage of MMI≥ 20mg/d than that in the subgroup with an initial dosage of MMI< 20mg/d (37.1% [23/62] *vs* 15.2% [9/59], P=0.006). After three months of treatment, the sTSH levels was improved (**Figure 3A**, **Supplementary Table 1**), while the increase in sTSH levels was greater in the XKC+MMI group compared to the MMI group (mean ± SD: 4.86 ± 11.70 *vs* 1.15 ± 5.16, median [95% CI]: 0.026 [0, 2.36] *vs* 0.005 [0, 0.093], unadjusted P=0.193), and the decrease in TRAb levels was significantly greater in the XKC+MMI group compared to the MMI group (mean ± SD: -3.94 ± 5.97 *vs* -1.06 ± 6.00, median [95% CI]: -2.66 [-5.19, -1.72] *vs* -0.945 [-1.48, -0.31], unadjusted P=0.030) (**Figure 3B**, **Supplementary Table 1**).

**Table 2:** Baseline characteristics of subgroup (n=121).

Variables	XKC+MMI (n=32)	MMI (n=89)	P
Male, n (%)	8 (25.0)	24 (27.0)	>0.999
Age, year, mean±SD	37.88±11.80	38.96±12.46	0.713
FT3, pmol/L			
N (N missing)	29 (3)	85 (4)	
mean±SD	16.56±14.88	13.71±12.03	0.684
Median (Q1, Q3)	10.07 (5.26, 25.88)	8.29 (5.32, 18.36)	
FT3, n (%)			0.359
Normal range	13 (40.6)	32 (36.0)	
Below normal		1 (1.1)	
Above normal	16 (50.0)	52 (58.4)	
Missing	3 (9.4)	4 (4.5)	
FT4, pmol/L			
N (N missing)	32 (0)	89 (0)	
mean±SD	44.4±35.86	36.95±29.75	0.542
Median (Q1, Q3)	29.65 (14.64, 81.47)	23.68 (16.10, 47.21)	
FT4, n (%)			0.949
Normal range	9 (28.1)	26 (29.2)	
Below normal	4 (12.5)	10 (11.2)	
Above normal	19 (59.4)	53 (59.6)	
Missing			
sTSH, uIU/L			
N (N missing)	32 (0)	89 (0)	
mean±SD	0.28±0.68	0.80±2.88	0.370
Median (Q1, Q3)	0.005 (0.005, 0.008)	0.005 (0.005, 0.019)	
sTSH, n (%)			0.426
Normal range	5 (15.6)	14 (15.7)	
Below normal	27 (84.4)	70 (78.7)	
Above normal	0 (0)	5 (5.6)	
TRAb, IU/L			
N (N missing)	31 (1)	80 (9)	
mean±SD	19.47±12.24	13.67±11.46	**0.018**
Median (Q1, Q3)	17.06 (10.01, 29.27)	10.50 (5.01, 19.45)	
TRAb, n (%)			0.070
Normal range	1 (3.1)	9 (10.1)	
Above normal	30 (93.8)	71 (79.8)	
Missing	1 (3.1)	9 (10.1)	
Initial dose of MMI, mg/d, n (%)			**0.012**
<20	9 (28.1)	50 (56.2)	
≥20	23 (71.9)	39 (43.8)	
Duration of XKC administration, month, n (%)			
1	4 (12.5)	–	
2	6 (18.8)	–	
3	22 (68.8)	–	

XKC, Xiakucao Oral Liquid; MMI, Methimazole; sTSH, sensitive thyroid-stimulating hormone; TRAb, thyroid-stimulating hormone receptor antibody; FT3, free triiodothyronine; FT4, free thyroxine.

Bold values indicate P<0.05.

At baseline, the number of patients with normal sTSH in XKC+MMI and MMI group were 5 (15.6%) and 14 (15.7%), respectively (**Table 2**). After 3 months of treatment, 9 (28.1%) patients in XKC+MMI group and 32 (36.0%) patients in MMI group had sTSH within the normal range and there was no statistically significant difference between groups (P=0.334) (**Figure 3A**, **Supplementary Table 2**). Proportion of the patients within normal range of TRAb, FT3 and FT4 were all increased at 3 months, while there was no statistically significant difference in the proportion of patients with normal FT3 or TRAb between the two groups at 3 months (**Supplementary Table 2**).

### 3.3 Univariate and multivariate regression analysis of subgroups

Regression analysis model 1 was performed to assess the association between the use of XKC and the change in sTSH in the third month. After adjusting for gender, age, baseline FT3, baseline FT4, baseline sTSH, and baseline TRAb, compared to MMI alone, treatment with XKC+MMI was found to be independently associated with an increase in sTSH levels at 3 months (multivariate: β=3.346, 95% CI: 0.353-6.339, P=0.031) (**Table 3**); the result of imputed data showed that XKC+MMI was associated with the increase of sTSH levels at 3 months (univariate: β=3.702, 95% CI: 0.695-6.709, P=0.017) (**Table 3**).

**Table 3:** Model 1: linear Regression models forassociated factors between sTSH CFB at 3 months in subgroup.

Characteristics	Non-imputed data				Imputed data			
	Univariate analysis		Multivariate analysis		Univariate analysis		Multivariate analysis	
	β (95% CI)	P	β (95% CI)	P*	β (95% CI)	P	β (95% CI)	P*
Male	0.068 (-3.011, 3.147)	0.966	0.147 (-2.832, 3.126)	0.923	0.068 (-3.011, 3.147)	0.966	-0.196 (-3.142, 2.75)	0.896
Age	-0.066 (-0.176, 0.044)	0.245	-0.054 (-0.168, 0.06)	0.357	-0.066 (-0.176, 0.044)	0.245	-0.062 (-0.174, 0.05)	0.275
FT3								
Normal range	(Referent)							
Below normal	1.91 (-12.59, 16.41)	0.797	1.494 (-13.553, 16.541)	0.846	1.91 (-12.782, 16.602)	0.799	1.35 (-13.666, 16.366)	0.86
Above normal	3.331 (0.575, 6.087)	**0.019**	1.121 (-3.344, 5.586)	0.624	3.884 (1.144, 6.624)	**0.006**	1.081 (-3.48, 5.642)	0.643
Missing	9.251 (3.424, 15.078)	**0.002**	5.998 (-1.103, 13.099)	0.101				
FT4								
Normal range	(Referent)							
Below normal	1.582 (-2.985, 6.149)	0.499	2.362 (-2.395, 7.119)	0.333	1.582 (-2.985, 6.149)	0.499	2.335 (-2.389, 7.059)	0.335
Above normal	4.504 (1.529, 7.479)	**0.004**	2.681 (-1.921, 7.283)	0.256	4.504 (1.529, 7.479)	**0.004**	2.445 (-2.175, 7.065)	0.302
sTSH								
Normal range	(Referent)							
Below normal	2.782 (-0.815, 6.379)	0.132	0.901 (-3.687, 5.489)	0.701	2.782 (-0.815, 6.379)	0.132	-0.14 (-4.746, 4.466)	0.953
Above normal	-7.644 (-14.849, -0.439)	**0.04**	-6.632 (-14.015, 0.751)	0.081	-7.644 (-14.849, -0.439)	**0.04**	-6.712 (-14.058, 0.634)	0.076
TRAb								
Normal range	(Referent)							
Above normal	1.01 (-3.957, 5.977)	0.691	-3.185 (-8.361, 1.991)	0.23	1.048 (-3.491, 5.587)	1.048	-3.476 (-8.276, 1.324)	0.159
Missing	1.663 (-5.038, 8.364)	0.628	-1.655 (-8.548, 5.238)	0.639				
XKC+MMI vs MMI	3.702 (0.695, 6.709)	**0.017**	3.346 (0.353, 6.339)	**0.031**	3.702 (0.695, 6.709)	**0.017**	2.828 (-0.251, 5.907)	0.075

CFB, change from baseline; XKC, Xiakucao Oral Liquid; MMI, Methimazole; sTSH, sensitive thyroid-stimulating hormone; TRAb, thyroid-stimulating hormone receptor antibody; FT3, free triiodothyronine; FT4, free thyroxine.

*, P value for adjusted analysis.

Bold values indicate P<0.05.

Regression analysis model 2 was used to assessed the association between the duration of XKC treatment and the change in sTSH in the third month. After adjusting for gender, age, baseline FT3, baseline FT4, baseline sTSH, baseline TRAb, and the initial dose of MMI, the three-month treatment with XKC+MMI was independently associated with an increase in sTSH levels at 3 months (multivariate: β=4.062, 95% CI: 0.516-7.608, P=0.027) (**Table 4**). And after imputation, the results also showed that three-month treatment with XKC+MMI was independently associated with an increase in sTSH levels at 3 months (multivariate: β=4.144, 95% CI: 0.600-7.688, P=0.024) (**Table 4**).

**Table 4:** Model 2: linear Regression models forassociated factors between sTSH CFB at 3 months in subgroup.

Characteristics	Non-imputed data				Imputed data			
	Univariate analysis		Multivariate analysis		Univariate analysis		Multivariate analysis	
	β (95% CI)	P	β (95% CI)	P*	β (95% CI)	P	β (95% CI)	P*
Male	0.068 (-3.011, 3.147)	0.966	0.209 (-2.792, 3.21)	0.892	0.068 (-3.011, 3.147)	0.966	0.004 (-2.985, 2.993)	0.998
Age	-0.066 (-0.176, 0.044)	0.245	-0.048 (-0.162, 0.066)	0.404	-0.066 (-0.176, 0.044)	0.245	-0.064 (-0.176, 0.048)	0.267
FT3								
Normal range	(Referent)							
Below normal	1.91 (-12.59, 16.41)	0.797	0.973 (-13.992, 15.938)	0.899	1.91 (-12.782, 16.602)	0.799	0.974 (-14.065, 16.013)	0.899
Above normal	3.331 (0.575, 6.087)	**0.019**	0.352 (-4.138, 4.842)	0.878	3.884 (1.144, 6.624)	**0.006**	0.822 (-3.747, 5.391)	0.725
Missing	9.251 (3.424, 15.078)	**0.002**	5.258 (-1.888, 12.404)	0.152				
FT4								
Normal range	(Referent)							
Below normal	1.582 (-2.985, 6.149)	0.499	2.648 (-2.144, 7.44)	0.281	1.582 (-2.985, 6.149)	0.499	2.544 (-2.244, 7.332)	0.3
Above normal	4.504 (1.529, 7.479)	**0.004**	2.214 (-2.384, 6.812)	0.347	4.504 (1.529, 7.479)	**0.004**	2.412 (-2.208, 7.032)	0.308
sTSH								
Normal range	(Referent)							
Below normal	2.782 (-0.815, 6.379)	0.132	0.299 (-4.458, 5.056)	0.902	2.782 (-0.815, 6.379)	0.132	0.025 (-4.65, 4.7)	0.992
Above normal	-7.644 (-14.849, -0.439)	**0.04**	-7.154 (-14.529, 0.221)	0.06	-7.644 (-14.849, -0.439)	**0.04**	-6.855 (-14.227, 0.517)	0.071
TRAb								
Normal range	(Referent)							
Above normal	1.01 (-3.957, 5.977)	0.691	-3.388 (-8.572, 1.796)	0.203	1.048 (-3.491, 5.587)	1.048	-3.153 (-7.969, 1.663)	0.202
Missing	1.663 (-5.038, 8.364)	0.628	-2.141 (-9.025, 4.743)	0.543				
Initial dose of MMI								
<20	(Referent)							
≥20	4.603 (2.016, 7.19)	**0.001**	2.21 (-1.108, 5.528)	0.195	4.603 (2.016, 7.19)	**0.001**	2.487 (-0.855, 5.829)	0.148
Treatment								
MMI alone	(Referent)							
MMI+XKC for 1 month	0.558 (-6.831, 7.947)	0.883	-0.272 (-7.698, 7.154)	0.943	0.558 (-6.831, 7.947)	0.883	0.628 (-6.728, 7.984)	0.867
MMI+XKC for 2 months	-0.665 (-6.763, 5.433)	0.831	-0.349 (-6.541, 5.843)	0.912	-0.665 (-6.763, 5.433)	0.831	-0.4 (-6.605, 5.805)	0.9
MMI+XKC for 3 months	5.465 (2.023, 8.907)	**0.002**	4.062 (0.516, 7.608)	**0.027**	5.465 (2.023, 8.907)	**0.002**	4.144 (0.600, 7.688)	**0.024**

CFB, change from baseline; XKC, Xiakucao Oral Liquid; MMI, Methimazole; sTSH, sensitive thyroid-stimulating hormone; TRAb, thyroid-stimulating hormone receptor antibody; FT3, free triiodothyronine; FT4, free thyroxine.

*, P value for adjusted analysis.

Bold values indicate P<0.05.

The further interaction analysis conducted to control for confounding factors revealed that there was no interaction between the initial dose of MMI and the groups in both the non-imputated and imputated data (all P>0.05) (**Supplementary Table 3**).

Regression analysis was conducted with the change in TRAb levels at 3 months as the outcome. After adjusting for gender, age, baseline FT3, baseline FT4, baseline sTSH, and baseline TRAb, compared with MMI alone, treatment with XKC+MMI showed a trend towards an association with a decrease in TRAb levels at 3 months (univariate: β=-2.888, 95% CI: -5.45–0.326, P=0.029; multivariate: β=-2.405, 95% CI: -4.794–0.016, P=0.051) (**Table 5**).

**Table 5:** Linear Regression models forassociated factors between TRAb CFB at 3 months in subgroup.

Characteristics	Univariate analysis		Multivariate analysis	
	β (95% CI)	P	β (95% CI)	P
Male	0.5 (-2.15, 3.15)	0.712	0.722 (-1.671, 3.115)	0.556
Age	0.104 (0.004, 0.204)	**0.044**	0.094 (-0.004, 0.192)	0.065
FT3				
Normal	(Referent)			
Below normal	-2.123 (-13.781, 9.535)	0.722	-4.641 (-16.093, 6.811)	0.429
Above normal	-2.467 (-4.835, -0.099)	**0.044**	-2.492 (-6.2, 1.216)	0.191
Missing	-7.962 (-13.003, -2.921)	**0.003**	-6.533 (-12.423, -0.643)	**0.032**
FT4				
Normal	(Referent)			
Below normal	-0.832 (-4.889, 3.225)	0.689	-0.226 (-4.144, 3.692)	0.91
Above normal	-1.603 (-4.22, 1.014)	0.233	3.115 (-0.678, 6.908)	0.111
sTSH				
Normal	(Referent)			
Below normal	-6.005 (-8.921, -3.089)	**<0.001**	-5.72 (-9.334, -2.106)	**0.003**
Above normal	-3.932 (-9.596, 1.732)	0.177	-6.151 (-11.762, -0.54)	**0.034**
TRAb				
Normal	(Referent)			
Above normal	-5.662 (-9.504, -1.82)	**0.005**	-2.62 (-6.556, 1.316)	0.195
XKC+MMI vs MMI	-2.888 (-5.45, -0.326)	**0.029**	-2.405 (-4.794, -0.016)	0.051

CFB, change from baseline; XKC, Xiakucao Oral Liquid; MMI, Methimazole; sTSH, sensitive thyroid-stimulating hormone; TRAb, thyroid-stimulating hormone receptor antibody; FT3, free triiodothyronine; FT4, free thyroxine.

Bold values indicate P<0.05.

## 4 Discussion

Our study found that the XKC combination treatment might promote early recovery of thyroid function. However, adherence to and persistence with XKC treatment still need to be improved.

In recent years, the advantages of traditional Chinese medicine (TCM) in the treatment of thyroid diseases have drawn attention. Many studies have demonstrated that the integration of TCM with western medical treatments for hyperthyroidism offers several advantages, such as shortening the course of treatment, reducing thyroid volume, alleviating symptoms, and reducing adverse reactions (1). Hyperthyroidism falls into the categories of “Ying Disease” and “Ying Qi” in TCM. XKC, which is characterized by its cold nature and bitter, pungent taste, is known for its effects of detoxify the liver and improve vision or eye health, dispersing liver dampness and alleviating depression, and dissipating phlegm and resolving masses. It is often used in clinical practice to treat scrofula, goiter, breast abscesses, swelling and pain, etc. Pharmacological studies have confirmed that XKC possesses significant benefits in terms of anti-inflammation, detumescence, antibacterial and immune regulation (32). The study found that *Prunella vulgaris* polysaccharide, one of the main active ingredients of XKC, can ameliorate the symptoms and thyroid function in a mouse model of GD mice. This improvement is potentially attributable to inhibitory effects on the phosphorylation of Raf, MEK1/2 and ERK1/2, as well as its regulatory role in Ras/Raf/mitogen-activated protein kinase/ERK signaling pathway. Additionally, it downregulates the expression of cytokines, including interferon γ, IL-6, IL-17 and TGF-β1 (33). A meta-analysis encompassing 8 randomized controlled studies with a total of 800 patients demonstrated that the combination of XKC and conventional Western medicine treatment could enhance clinical outcomes for hyperthyroidism compared with Western medicine. This combination therapy was found to improve clinical efficacy, reduce the levels of FT3, FT4 and TRAb, reduce the volume of goiter, and increase the level of TSH (34). However, the findings from this single-center study showed that the clinical application of XKC was insufficient, and only about 25% of GD patients were treated with XKC. It is necessary to further improve the popularization of XKC application in clinical practice and to fully exploit the advantages of TCM in the treatment of GD.

TCM treatment for GD emphasizes “syndrome differentiation and treatment”, which advocates for personalized treatment tailored to the specific symptoms, constitutions and causes of patients. The duration of the treatment course and the consistency of medication adherence play a crucial role in patient recovery and return to a state of good health. The etiology of GD can be attributed to several aspects, including congenital endowment deficiency, acquired emotional damage, and external pathogen invasion, all of which lead to the imbalance of yin and yang, qi and blood, as well as the dysfunction of zang-fu organs (35). At different stages of the disease course, the focus of syndrome differentiation and treatment is also different. At the early stage of the disease, qi stagnation transforming into fire and giving rise to phlegm is the main manifestation. In the middle stage, both deficiency and excess syndromes are present, and equal importance is attached to phlegm stasis and yin deficiency. In the later stage, qi and yin deficiency is predominant, accompanied by the obstruction of phlegm and stasis (36). The treatment process usually requires long-term, continuous and stable medication in order to gradually adjust the balance of the body. However, the results of this single-center study showed that the clinical compliance with continuous medication for XKC was rather poor and only 13.3% of the patients were treated with XKC continuously for 3 months. If GD patients could receive long-term, continuous, and stable combined treatment with XKC, it might have a more favorable impact on the treatment outcome.

This study found that the percentage of patients receiving XKC+MMI was significantly higher in patients with an initial dosage of MMI≥20mg/d than those with an initial dosage of MMI<20mg/d (35.1% *vs* 19.6%, P<0.001). The initial dose of MMI is generally 10–30 mg/d. However, for patients with severe symptoms and significant elevation in thyroid hormones levels, a higher dose of MMI is required (2). However, the higher the dose of MMI is, the more likely it will cause adverse reactions such as skin problems (37), agranulocytosis (38, 39), and severe liver injury (40). A study involving 100 GD patients showed that XKC+MMI and MMI treatment alone for 1 year (initial dose of MMI was 30–45 mg) resulted in different degrees of adverse reactions, mainly involving leukopenia, abnormal liver function, and secondary hypothyroidism caused by long-term use of MMI. However, the incidence of the above adverse reactions was significantly lower in the XKC+MMI group compared to the group with MMI alone (6% *vs* 36%, P<0.01) (23). Therefore, we hypothesize that for patients with a relatively high initial dose of MMI, the more use of XKC+MMI treatment is related to the fact that the combination of TCM with MMI can alleviate relevant adverse reactions. In addition, this study found that the percentage of patients with suppressed sTSH levels, below the normal range, was higher in patients treated with XKC+MMI than in those with MMI alone (81.4% *vs* 69.5%, P=0.01). The lower the initial test results of sTSH, the more serious is the disease. The XKC+MMI combination may exert a synergistic effect on the improvement of FT3, FT4, TSH and TRAb levels in the treatment of GD (1), which may explain why patients with low initial test results of sTSH are more likely to receive XKC+MMI treatment. More studies are needed to confirm the above speculation.

This study explored the association between the treatment of XKC+MMI and the early recovery of thyroid function in GD patients. It was found that the XKC+MMI regimen was superior to MMI alone in increasing the sTSH levels at 3 months, especially when XKC+MMI was used in combination for 3 months. In addition, XKC+MMI tended to be superior to MMI alone in reducing TRAb levels at 3 months. Consistent with these results, previous studies have also suggested that the use of XKC+MMI in the treatment of GD can promote the early improvement of thyroid function. A randomized controlled trial involving 120 patients with GD found that XKC+MMI treatment could improve early thyroid function compared to MMI alone. This was evidenced by a significant increase in TSH levels during the first and second months (0.11 ± 0.09 *vs* 0.18 ± 0.10, P<0.05; 0.20 ± 0.13 *vs* 0.31 ± 0.22, P<0.05) and a decrease in FT3 and FT4 levels during the same periods (9.03 ± 3.47 *vs* 7.17 ± 2.85, P<0.05; 7.22 ± 1.83 *vs* 5.97 ± 1.26, P<0.05; 30.26 ± 7.49 *vs* 24.61 ± 5.82, P<0.05; 20.17 ± 6.02 *vs* 17.75 ± 5.23, P<0.05) (24). In addition, when XKC was integrated with conventional Western medicine treatment regimens (propylthiouracil and MMI) for 12 weeks, it was beneficial in improving the clinical symptoms and signs of GD patients and reducing the size of goiter, which may be related to the reduction in TRAb levels (41). Further large-scale, prospective studies are needed to verify the findings of this study in the future.

In subgroup analysis, although the change of sTSH was higher in XKC+MMI group, there was no significant difference in the proportion of patients with normal sTSH and TRAb between two groups. The lack of statistical significance in euthyroid rates between groups may stem from sample size limitation and poor compliance of treatment, approximately one-third of the patients did not receive continuous treatment with XKC. Previous randomized trials have demonstrated that combining XKC with MMI achieves significant improvements of thyroid hormone level in GD patients within 3 months (24, 41).These findings align with our results, where XKC+MMI showed a clinically meaningful (albeit non-significant) trend toward TRAb reduction and significant sTSH elevation at 3 months. The lack of statistical significance in TRAb reduction may reflect suboptimal adherence to XKC.

This study has the some limitations. First, retrospective analysis of real-world data and missing data can lead to potential selection bias. As a retrospective analysis of real-world clinical practice data, this study is susceptible to selection bias, particularly in patient enrollment and treatment allocation. To mitigate this risk, we included all consecutive patients meeting the inclusion/exclusion criteria and employed multiple imputation to address missing data. These strategies aimed to enhance representativeness and reduce bias. However, residual confounding from unmeasured variables may persist, a limitation inherent to observational studies. Second, the small sample size might lead to sampling error and limited statistical power. The relatively small sample size, especially in subgroup analyses (e.g., XKC+MMI *vs*. MMI subgroups), may introduce sampling error and reduce statistical power. Notably, the subgroup proportions (1:3 for XKC+MMI: MMI) aligned with the initial screening population, reflecting real-world clinical distributions. A *post hoc* power analysis based on TRAb reduction revealed suboptimal power, underscoring the need for larger cohorts in future studies. We plan to expand recruitment to validate findings and improve precision. Third, the missing data might cause bias. Missing data, particularly in laboratory parameters, were inevitable in real-world settings. To address this, we applied multiple imputation using chained equations (MICE) and performed sensitivity analyses comparing imputed and complete-case datasets. The consistency in effect estimates (e.g., XKC+MMI association with sTSH reduction) across both datasets supports the robustness of our primary conclusions. Forth, confounding bias caused by heterogeneity in baseline disease severity. Baseline differences in disease severity could confound outcomes. To evaluate this, we tested interaction terms between treatment group and MMI initial dose (a proxy for disease severity). The absence of significant interaction effects suggests that disease severity did not modify the treatment effect. Nonetheless, residual confounding from unmeasured severity indicators (e.g., thyroid volume) cannot be fully excluded. Fifth, 3-month XKC+MMI treatment might not enough to normalize thyroid functions. While 3-month XKC+MMI therapy did not fully restore euthyroidism, its superiority in sTSH improvement underscores its potential as an adjunctive strategy. Enhancing adherence through targeted interventions could unlock greater clinical benefits, warranting further investigation in pragmatic trials. In the future, a prospective cohort study with a large sample size will be conducted to further explore the impact of long-term, regular use of XKC in the treatment of GD on prognosis.

## 5 Conclusion

In clinical practice, approximately one-quarter of GD patients receive combination treatment with XKC, and only a small number of patients are able to continue taking it for more than two months. GD patients with a higher initial dose of MMI and a lower initial sTSH level are more likely to receive combination treatment of XKC+MMI. The clinical application and patient compliance of XKC need to be improved to make full use of its advantages. This study explored the association between the treatment of XKC+MMI and the early recovery of thyroid function in GD patients. The results indicate that the a three-month treatment of MMI+XKC is an independent factor for the increase in sTSH levels at 3 months. XKC+MMI treatment appears to promote the early improvement of thyroid function in GD patients. Further large-scale, prospective studies are needed for in-depth exploration.

## Data Availability

The original contributions presented in the study are included in the article/**Supplementary Material**. Further inquiries can be directed to the corresponding author.

## References

[1] Standardization project group of Clinical Application Guidelines for the Treatment of Dominant Diseases with Chinese Patent Medicine. Clinical application guidelines for the adjuvant treatment of Graves’ disease with Chinese patent medicine (2021). Chin J Integrated Traditional Western Medicine. (2022) 42:1029–39. doi: 10.7661/j.cjim.20220610.079

[2] Chinese Society of Endocrinology CMA Chinese Society of Endocrinology and Metabolism Physicians Chinese Medical Doctor Association Chinese Society of Nuclear Medicine, Chinese Society of Thyroid and Metabolic Surgery Committee of Thyroid Surgeons Chinese Society of Surgeons Chinese Medical Doctor Association. Guidelines for diagnosis and management of hyperthyroidism and other causes of thyrotoxicosis. Int J Endocrinol Metab. (2022) 42:401–50. doi: 10.3760/cma.j.cn311282-20220624-00404-1

[3] Zhou F Wang X Wang L Sun X Tan G Wei W. Genetics, epigenetics, cellular immunology, and gut microbiota: emerging links with graves’ Disease. Front Cell Dev Biol. (2021) 9:794912. doi: 10.3389/fcell.2021.794912 35059400 PMC8765724

[4] Nyirenda MJ Clark DN Finlayson AR Read J Elders A Bain M. Thyroid disease and increased cardiovascular risk. Thyroid. (2005) 15:718–24. doi: 10.1089/thy.2005.15.718 16053389

[5] Brandt F Almind D Christensen K Green A Brix TH Hegedüs L. Excess mortality in hyperthyroidism: the influence of preexisting comorbidity and genetic confounding: a danish nationwide register-based cohort study of twins and singletons. J Clin Endocrinol Metab. (2012) 97:4123–9. doi: 10.1210/jc.2012-2268 PMC3485592 22930783

[6] Lillevang-Johansen M Abrahamsen B Jørgensen HL Brix TH Hegedüs L. Excess mortality in treated and untreated hyperthyroidism is related to cumulative periods of low serum TSH. J Clin Endocrinol Metab. (2017) 102:2301–9. doi: 10.1210/jc.2017-00166 28368540

[7] Naser JA Pislaru S Stan MN Lin G. Incidence, risk factors, natural history and outcomes of heart failure in patients with Graves’ disease. Heart. (2022) 108:868–74. doi: 10.1136/heartjnl-2021-319752 34489313

[8] Dekkers OM Horváth-Puhó E Cannegieter SC Vandenbroucke JP Sørensen HT Jørgensen JO. Acute cardiovascular events and all-cause mortality in patients with hyperthyroidism: a population-based cohort study. Eur J Endocrinol. (2017) 176:1–9. doi: 10.1530/eje-16-0576 27697972

[9] Folkestad L Brandt F Lillevang-Johansen M Brix TH Hegedüs L. Graves’ Disease and toxic nodular goiter, aggravated by duration of hyperthyroidism, are associated with alzheimer’s and vascular dementia: A registry-based long-term follow-up of two large cohorts. Thyroid. (2020) 30:672–80. doi: 10.1089/thy.2019.0672 31984866

[10] Lillevang-Johansen M Abrahamsen B Jorgensen HL Brix TH Hegedus L. Duration of hyperthyroidism and lack of sufficient treatment are associated with increased cardiovascular risk. Thyroid. (2019) 29:332–40. doi: 10.1089/thy.2018.0320 30648498

[11] Okosieme OE Taylor PN Evans C Thayer D Chai A Khan I. Primary therapy of Graves’ disease and cardiovascular morbidity and mortality: a linked-record cohort study. Lancet Diabetes Endocrinol. (2019) 7:278–87. doi: 10.1016/s2213-8587(19)30059-2 30827829

[12] Shahida B Tsoumani K Planck T Modhukur V Asp P Sundlov A. Increased risk of Graves ophthalmopathy in patients with increasing TRAb after radioiodine treatment and the impact of CTLA4 on TRAb titres. Endocrine. (2022) 75:856–64. doi: 10.1007/s12020-021-02952-2 PMC8888513 34859391

[13] Lantz M Planck T Asman P Hallengren B. Increased TRAb and/or low anti-TPO titers at diagnosis of graves’ disease are associated with an increased risk of developing ophthalmopathy after onset. Exp Clin Endocrinol Diabetes. (2014) 122:113–7. doi: 10.1055/s-0033-1363193 24554511

[14] Hamada N Momotani N Ishikawa N Yoshimura Noh J Okamoto Y Konishi T. Persistent high TRAb values during pregnancy predict increased risk of neonatal hyperthyroidism following radioiodine therapy for refractory hyperthyroidism. Endocr J. (2011) 58:55–8. doi: 10.1507/endocrj.k10e-123 20962435

[15] Priyanka R Sridhar S Sumathi B Jeyaraj AR Natarajan V Subbiah E. Third-Generation Thyrotropin Receptor Antibody (TRAb) assay for predicting neonatal thyroid dysfunction in pregnant women with Graves’ disease. Endocrine. (2024) 84:500–8. doi: 10.1007/s12020-023-03569-3 37861945

[16] Li D Pei H Li X Liu X Li X Xie Y. Short-term effects of combined treatment with potassium bromide and methimazole in patients with Graves’ disease. J Endocrinol Invest. (2012) 35:971–4. doi: 10.3275/8188 22186223

[17] Benker G Vitti P Kahaly G Raue F Tegler L Hirche H. Response to methimazole in Graves’ disease. The European Multicenter Study Group. Clin Endocrinol (Oxf). (1995) 43:257–63. doi: 10.1111/j.1365-2265.1995.tb02030.x 7586593

[18] Nakamura H Noh JY Itoh K Fukata S Miyauchi A Hamada N. Comparison of methimazole and propylthiouracil in patients with hyperthyroidism caused by Graves’ disease. J Clin Endocrinol Metab. (2007) 92:2157–62. doi: 10.1210/jc.2006-2135 17389704

[19] Lee SY Pearce EN. Hyperthyroidism: A review. Jama. (2023) 330:1472–83. doi: 10.1001/jama.2023.19052 PMC10873132 37847271

[20] Sjölin G Holmberg M Törring O Byström K Khamisi S de Laval D. The long-term outcome of treatment for graves’ Hyperthyroidism. Thyroid. (2019) 29:1545–57. doi: 10.1089/thy.2019.0085 31482765

[21] Azizi F Amouzegar A Tohidi M Hedayati M Khalili D Cheraghi L. Increased remission rates after long-term methimazole therapy in patients with graves’ Disease: results of a randomized clinical trial. Thyroid. (2019) 29:1192–200. doi: 10.1089/thy.2019.0180 31310160

[22] Zhang Q Chi L Zhou B Xu H. To observe the prognosis of ATD on graves syndrome and the relationship with TSH in the serum. West China Med J. (2006) 21:2. doi: 10.3969/j.issn.1002-0179.2006.03.012

[23] Chai L Wang J Wei Y Liu C. Clinical observation of Prunella vulgaris oral liquid combined with metimidazole in the treatment of syndrome of hyperactivity of heart and liver fire of Graves’ disease. Chin J Hosp Pharmacy. (2020) 40:1246–51. doi: 10.13286/j.1001-5213.2020.11.15

[24] Wu S. Clinical effect of Xiakucao Oral Liquid on the Graves disease. Chin Traditional Patent Medicine. (2012) 34:3. doi: 10.3969/j.issn.1001-1528.2012.01.004

[25] Qin S Kong F Liang Y Yang B Zhang X Wang P. Efficacy of Yikang powder combined with tapazol in the patients with Graves disease. Chin J New Drugs. (2010) 19:39–41,55.

[26] Li H Zhang Z Cheng B Yin L Zhao H Li Z. Clinical observation of sanjie jiaxiao prescription and small dose of thyrozol in treatment of 60 cases of graves disease. Liaoning J Traditional Chin Medicine. (2015) 42:750–3. doi: 10.13192/j.issn.1000-1719.2015.04.033

[27] Wang Y Han D Zheng X. Effect of xiakucao granules combined with thiamazole on the autoantibody and cytokines in patients with graves disease. Genomics Appl Biol. (2017) 36:3635–41. doi: 10.13417/j.gab.036.0003635

[28] Han Z Cen C Ou Q Pan Y Zhang J Huo D. The potential prebiotic berberine combined with methimazole improved the therapeutic effect of graves’ Disease patients through regulating the intestinal microbiome. Front Immunol. (2021) 12:826067. doi: 10.3389/fimmu.2021.826067 35082799 PMC8785824

[29] Xie Y Deng B Huang X Wen C Jie Y. Effects of Xiakucao oral liquid on thyroid size and thyroid stimulating receptor antibody in patients with Graves’ disease. Guangdong Med J. (2015) 36:311–3. doi: 10.13820/j.cnki.gdyx.2015.02.029

[30] Chen X. Clinical observation on the treatment of toxic diffuse goiter disease with Xiakucao oral liquid combined with methimazole tablets. J Pract Traditional Chin Medicine. (2023) 39:2162–4.

[31] Chinese Medical Association Journal of the Chinese Medical Association Chinese Society of General Medicine Editorial Committee of Chinese Journal of General Practitioners Chinese Medical Association. Expert group for the development of guidelines for primary diagnosis and treatment of endocrine diseases. Guideline for primary care of hyperthyroidism (2019). Chin J Gen Practitioners. (2019) 18:1118–28. doi: 10.3760/cma.j.issn.1671-7368.2019.12.002

[32] Li D Niu M Zhang J. Effect of Xiakucao oral liquid on STAT3 and NF-KB signal transduction pathway. Zhejiang Clin Med J. (2019) 21:323–5.

[33] Xiang J Wang B Wang Y Xu W. Improvement effect and its mechanism of Prunella vulgaris polysaccharides on Graves’disease mice. Guangxi Med J. (2020) 42:1850–4. doi: 10.11675/j.issn.0253-4304.2020.14.19

[34] Zhu X Fan Y Zhang H Sun H Cao L Cao W. Systemic review of Prunella vulgaris Oral Liquid in treatment of hyperthyroidism. Drug Eval Res. (2021) 44:1764–71. doi: 10.7501/j.issn.1674-6376.2021.08.031

[35] Chen X Wang L Liu Z Ren K Qi G. Summary of experience in treating hyperthyroidism with TCM syndrome differentiation. JBM. (2022) 10:167–73. doi: 10.4236/jbm.2022.109013

[36] Chen J Xiao W. Pathogenesis and treatment of hyperthyroidism. Hunan J Traditional Chin Medicine. (2012) 28:78–9. doi: 10.16808/j.cnki.issn1003-7705.2012.02.051

[37] Otsuka F Noh JY Chino T Shimizu T Mukasa K Ito K. Hepatotoxicity and cutaneous reactions after antithyroid drug administration. Clin Endocrinol (Oxf). (2012) 77:310–5. doi: 10.1111/j.1365-2265.2012.04365.x 22332800

[38] Takata K Kubota S Fukata S Kudo T Nishihara E Ito M. Methimazole-induced agranulocytosis in patients with Graves’ disease is more frequent with an initial dose of 30 mg daily than with 15 mg daily. Thyroid. (2009) 19:559–63. doi: 10.1089/thy.2008.0364 19445623

[39] Andres E Kurtz JE Perrin AE Dufour P Schlienger JL Maloisel F. Haematopoietic growth factor in antithyroid-drug-induced agranulocytosis. QJM. (2001) 94:423–8. doi: 10.1093/qjmed/94.8.423 11493719

[40] Wang MT Lee WJ Huang TY Chu CL Hsieh CH. Antithyroid drug-related hepatotoxicity in hyperthyroidism patients: a population-based cohort study. Br J Clin Pharmacol. (2014) 78:619–29. doi: 10.1111/bcp.12336 PMC4243912 25279406

[41] Zou Y. Effect of Xiukucao oral liquid on thyroid size and thyrotropin receptor antibody in patients with Graves’ disease. Modern J Integrated Traditional Chin Western Medicine. (2016) 25:2711–3. doi: 10.3969/j.issn.1008-8849.2016.24.033

