# Supplementary material for: Effectiveness of Xiakucao Oral Liquid combined with methimazole on early thyroid function recovery and treatment compliance in Graves’ disease: a real-world retrospective cohort study
Source: Front Endocrinol (Lausanne). 2025 Jun 6;16:1560262. doi: 10.3389/fendo.2025.1560262 (PMC12184096; doi:10.3389/fendo.2025.1560262)
Supplement: Supplementary file 1 [file DataSheet1.docx]

Supplementary Table 1. Comparison of thyroid hormones in subgroup (n=121)

| Variables | XKC+MMI (n=32) | MMI (n=89) | P |
| --- | --- | --- | --- |
| sTSH, uIU/L |  |  |  |
| Mean±SD |  |  |  |
| Baseline | 0.28 ± 0.68 | 0.80 ± 2.88 | 0.684 |
| 1st month | 0.13 ± 0.30 | 0.39 ± 1.27 | 0.710 |
| 2nd month | 2.30 ± 6.25 | 0.77 ± 2.14 | 0.920 |
| 3rd month | 5.13 ± 11.59 | 1.95 ± 4.33 | 0.718 |
| 3rd month - baseline | 4.86 ± 11.7 | 1.15 ± 5.16 | 0.193 |
| Median (95% CI) |  |  |  |
| 3rd month - baseline | 0.026 (0, 2.36) | 0.005 (0, 0.093) |  |
| TRAb, IU/L |  |  |  |
| Mean±SD |  |  |  |
| Baseline | 19.47 ± 12.24 | 13.67 ± 11.46 | 0.542 |
| 1st month | 21.16 ± 11.79 | 14.59 ± 12.21 | 0.148 |
| 2nd month | 15.35 ± 12.05 | 17.58 ± 12.30 | 0.814 |
| 3rd month | 15.12 ± 11.78 | 12.72 ± 10.64 | 0.153 |
| 3rd month - baseline | -3.94 ± 5.97 | -1.06 ± 6.00 | 0.030 |
| Median (95% CI) |  |  |  |
| 3rd month - baseline | -2.66 (-5.19, -1.72) | -0.945 (-1.48, -0.31) |  |
| FT3, pmol/L |  |  |  |
| Mean±SD |  |  |  |
| Baseline | 16.45 ± 14.88 | 13.71 ± 12.03 | 0.370 |
| 1st month | 8.33 ± 5.21 | 8.08 ± 3.67 | 0.901 |
| 2nd month | 5.75 ± 2.03 | 6.53 ± 5.44 | 0.580 |
| 3rd month | 5.62 ± 2.19 | 6.64 ± 6.06 | 0.327 |
| 3rd month - baseline | -10.74 ± 15.22 | -7.25 ± 13.89 | 0.646 |
| Median (95% CI) |  |  |  |
| 3rd month - baseline | -4.06 (-11.28, -0.48) | -2.77 (-6.25, -1.52) |  |
| FT4, pmol/L |  |  |  |
| Mean±SD |  |  |  |
| Baseline | 44.44 ± 35.86 | 36.95 ± 29.75 | 0.176 |
| 1st month | 18.54 ± 10.17 | 21.26 ± 9.54 | 0.033 |
| 2nd month | 13.11 ± 5.63 | 15.84 ± 12.79 | 0.604 |
| 3rd month | 13.18 ± 6.21 | 16.69 ± 12.68 | 0.249 |
| 3rd month - baseline | -31.26 ± 38.81 | -20.26 ± 32.54 | 0.319 |
| Median (95% CI) |  |  |  |
| 3rd month - baseline | -19.98 (-39.14, -0.95) | -11.74 (-17.08, -4.045) |  |

Abbreviations: XKC, Xiakucao Oral Liquid; MMI, Methimazole; sTSH, sensitive thyroid-stimulating hormone; TRAb, thyroid-stimulating hormone receptor antibody; FT3, free triiodothyronine; FT4, free thyroxine.

Supplementary Table 2. Comparison of number of patients with normal thyroid hormones at 3 months in subgroup (n=121)

| Variables | XKC+MMI (n=32) | MMI (n=89) | P |
| --- | --- | --- | --- |
| sTSH, n (%) |  |  | 0.344 |
| Normal range | 9 (28.1) | 32 (36.0) |  |
| Below normal | 15 (46.9) | 46 (51.7) |  |
| Above normal | 8 (25.0) | 11 (12.4) |  |
| TRAb, n (%) |  |  | 0.784 |
| Normal range | 3 (9.4) | 10 (11.2) |  |
| Above normal | 27 (84.4) | 74 (83.2) |  |
| Missing | 2 (6.2) | 5 (5.6) |  |
| FT3, n (%) |  |  | >0.999 |
| Normal range | 24 (75.0) | 67 (75.3) |  |
| Below normal | 1 (3.1) | 4 (4.5) |  |
| Above normal | 7 (21.9) | 18 (20.2) |  |
| FT4, n (%) |  |  | 0.029 |
| Normal range | 13 (40.6) | 46 (51.7) |  |
| Below normal | 17 (53.1) | 29 (32.6) |  |
| Above normal | 2 (6.3) | 14 (15.7) |  |

Abbreviations: XKC, Xiakucao Oral Liquid; MMI, Methimazole; sTSH, sensitive thyroid-stimulating hormone; TRAb, thyroid-stimulating hormone receptor antibody; FT3, free triiodothyronine; FT4, free thyroxine.

Supplementary Table 3. Interaction testing between groups and initial dose of MMI for the CFB of sTSH at 3 months in subgroup (n=121)

| Interaction term | Model | Multiple imputation or not | P-value for interaction |
| --- | --- | --- | --- |
| Group × MMI dose | Model A | yes | 0.299 |
| Group × MMI dose | Model A | no | 0.299 |
| Group × MMI dose | Model B | yes | 0.203 |
| Group × MMI dose | Model B | no | 0.135 |
| XKC use time × MMI dose | Model C | yes | 0.102 |
| XKC use time × MMI dose | Model C | no | 0.108 |

Model A: tested interactions between group and MMI dose.

Model B: tested interactions between group and MMI dose were adjusted age, sex, baseline of TSH, baseline of TRAb, baseline of FT3, and baseline of FT4.

Model C: tested interactions between XKC use time and MMI dose were adjusted age, sex, baseline of TSH, baseline of TRAb, baseline of FT3, and baseline of FT4.
